# Tryambak Mahadev Gogate (1913-1998): A Pioneer of Panchakarma Therapies of Ayurveda

**DOI:** 10.7759/cureus.69597

**Published:** 2024-09-17

**Authors:** Satyajit P Kulkarni, Shivanand S Tonde

**Affiliations:** 1 Fivefold Bio Purification Therapy (Panchakarma), Manjushree Research Institute of Ayurvedic Science, Gandhinagar, IND; 2 Medicine, Datta Meghe Institute of Higher Education and Research, Wardha, IND; 3 Preventive Medicine and Yoga (Swasthavritta and Yoga), Ayurved Seva Sangh Ayurved Mahavidyalaya, Nashik, IND

**Keywords:** acute on chronic liver disease, ayurvedic medicine, diagnostic methods, tm gogate, vomitting

## Abstract

Tryambak Mahadev Gogate (1913-1998) was instrumental in the development of practical applications of Panchakarma therapies as well as in advancing Ayurvedic education and practice, which faced significant challenges during the colonial period in India. A gifted young individual, motivated by a desire to contribute to his nation, dedicated himself to the revitalization of Ayurveda. In pursuit of this goal, he engaged in self-experimentation, often at great personal risk. He meticulously documented his findings throughout these experiments, establishing techniques for administering Panchakarma therapies, refining Ayurvedic diagnostic methods, and contributing to the evolution of Ayurveda as a scientific discipline, all while making detailed observations that paralleled advancements in modern medical science.

## Introduction and background

Ayurveda, the ancient medical system of India, has undergone a remarkable journey of transformation throughout its rich history. This holistic approach, which focuses on health maintenance and disease treatment, has weathered numerous challenges, including the destruction of its texts and medicinal practices during India's encounters with foreign invasions. Despite these setbacks, Ayurveda has demonstrated remarkable resilience, adapting and evolving to meet the changing needs of the times [1].

One of the significant challenges faced by Ayurveda during the colonial period was the British government's prohibition of Ayurvedic schools, which they claimed were promoting anti-government sentiments. In response to this situation, it became imperative for an individual to step forward and champion the teaching and dissemination of Ayurveda [2,3]. Tryambak Mahadev Gogate demonstrated remarkable courage by initiating his studies in Ayurveda during this tumultuous time, overcoming numerous obstacles to ensure the preservation and propagation of this ancient medical tradition [4].

Ayurvedic treatment encompasses two primary elements: Panchakarma and Ayurvedic medicines. Panchakarma consists of specific procedures designed to eliminate Doshas, thereby facilitating the removal of toxins from the body. Following these procedures, Ayurvedic medicines are administered to enhance their therapeutic effects. Panchakarma contributes to the promotion of overall health and the prevention of diseases [5,6].

However, the practical application of Panchakarma, particularly the Vamana (induced vomiting) procedure, faced challenges during this period. Dr. Gogate encountered numerous obstacles in executing Vamana, prompting him to seek information from various sources, gather necessary medicines, and ultimately overcome these challenges [4].

## Review

Early life

He was born on August 4, 1913. His father served as a royal physician within the Javhar dynasty in the state of Maharashtra. From his father, he inherited a foundational understanding of Ayurveda. In his youth, he exhibited a mischievous disposition. As he matured, he became inspired by the independence movement and, as previously noted, aspired to dedicate himself to the revolutionary cause. Subsequently, he developed a desire to study and teach Ayurveda, recognizing it as an indigenous science [4].

He enrolled at the G.S. Gune Ayurved College in Ahmednagar. He recounted an incident in which police conducted a raid on their school. He possessed several pamphlets related to the independence movement, which the police were specifically seeking. In a moment of quick thinking, Dr. Gogate swiftly disposed of the pamphlets by throwing them into the furnace where medicines were being prepared. This decisive action allowed him and his peers to evade capture, demonstrating his prudent nature [4].

His career

Following the completion of his Ayurved Teerth degree, Dr. Gogate traveled extensively before ultimately establishing his practice in Amaravati, a town located in the Vidarbha region of Maharashtra in 1934, during which he treated thousands of patients, adhering to Ayurvedic principles. His approach consistently prioritized the application of Panchakarma as a preliminary step. In addition to his private practice, he served as an anesthetic expert at the government hospital in Amravati, a role that elicited admiration and envy from his peers in the community. His wife, a practitioner of modern medicine specializing in obstetrics, complemented his Ayurvedic practice. Dr. Gogate firmly believed in the necessity of Panchakarma within Ayurvedic treatment, questioning the reluctance of some practitioners to adopt this foundational principle, as emphasized by ancient sages such as Charaka, Sushruta, and Vagbhata [5]. He was innovative in developing instruments for Panchakarma and in creating remedies for any complications that arose during treatment. Panchakarma constituted the core of his practice, and he held the Ashtang Hridya in high regard, frequently referencing it in his work. His profound respect for Vagbhata was evident; during challenging situations, he would recall verses from the Ashtang Hridya that conveyed the importance of remaining steadfast in the pursuit of righteousness, which provided him with significant mental fortitude. Throughout his career, Dr. Gogate demonstrated remarkable courage, often treating critically ill patients while first testing new procedures on himself [4].

He maintained transparency with his patients and their families, informing them of his intent to employ novel methods rather than conventional treatments. Only with the patient's consent would he proceed with such treatments, valuing their perspectives on their health and care. His extensive collection of patient insights regarding their illnesses and treatments was later published by Dr. T.M. Gogate Pratishthan in Nasik as a book titled "Panchakarma ki Satyakathaein" in 2010 [4].

Perspective building work

In the 1950s and 1960s, Ayurvedic practitioners frequently expressed skepticism about modern science. There were two prevailing perspectives: one advocated for the integration of Ayurveda with contemporary medical practices, while the other favored a strictly traditional approach to Ayurveda, eschewing modern medicine entirely. Dr. Gogate argued for a balanced integration of Ayurveda and modern medicine, emphasizing the importance of adhering to Ayurvedic principles without compromise [7].

Pioneer of Panchakarma equipment

Basti, an enema therapy and integral component of Panchakarma, demonstrates therapeutic benefits for various conditions, including constipation, lower back pain, and hemorrhoids. However, despite its therapeutic potential, the widespread adoption of Basti has been historically limited. This is likely attributable to the materials traditionally described for instrumentation in classical Ayurvedic texts, such as animal bladders and metallic nozzles, which may reflect the limited availability of suitable elastic materials in earlier eras. The cumbersome nature of these traditional instruments likely posed a barrier to practitioners, leading to a decline in the practical application of Basti [8].

Dr. Gogate, recognizing the need for modernization and drawing upon his knowledge of contemporary medical devices, introduced innovative modifications to Basti administration. He replaced traditional instruments with readily available and user-friendly alternatives, such as rubber catheters and syringes. Furthermore, he adopted the use of enema pots, commonly employed in midwifery during that period, for the administration of Basti. These adaptations significantly improved the practicality and ease of Basti administration, potentially contributing to its resurgence in Ayurvedic practice [4].

There existed a notable lack of awareness concerning contemporary research and innovations in modern medical science among Indian physicians, particularly those practicing Ayurveda. In contrast, Dr. Gogate demonstrated a commitment to staying informed about advancements in modern medical science. This was illustrated by his references to the 'Journal of Celone' and another journal from the UK. In his discussion on the efficacy of Ayurvedic purgatives in treating psychiatric disorders, he cited a retrospective study conducted in the UK that examined the prevalence of constipation in cases of suicide. The findings of this study indicated a significant correlation between constipation and suicidal tendencies [4].

In 1991, he received the prestigious Annasaheb Patwardhan Award and was elected chairperson of the Prantik Vaidya Mandal in 1964. Prior to these achievements, he represented the Indo-Russian Cultural Society during visits to Russia and the United States. While in Russia, he toured numerous medical colleges, where he delivered lectures on Ayurveda both in person and via radio broadcasts. Subsequently, he traveled to the United States and Brazil, promoting Ayurveda through the Maharshi Yogi Mission [4].

Dr. Gogate's contributions to the field of Ayurveda

Dr. Gogate’s contributions were multifaceted and significant. He demonstrated remarkable courage in championing the practice of Ayurveda during a period when it faced significant challenges, including the colonial government's prohibition of Ayurvedic schools [4].

**Figure 1:**
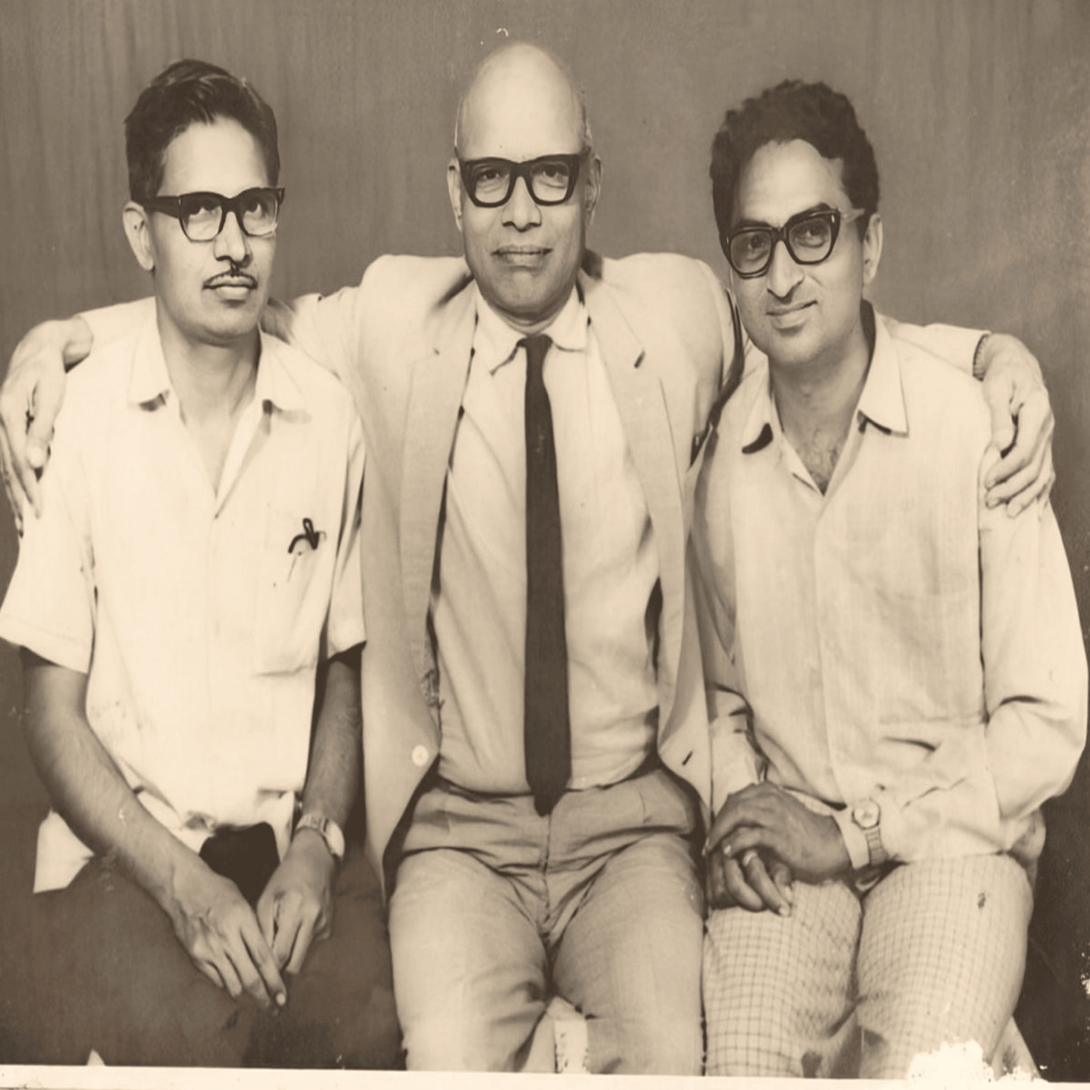
TM Gogate with his students S Girase (his left) and PT Joshi (his right) The original hard copy of this photograph is in the possession of ST, the second author of this manuscript. This photograph was previously published in two Marathi books that were edited and published by Tryambak Mahadev Pratishthan Nashik, an organization operated by ST.

He educated numerous students and motivated them to pass on their knowledge to others. Consequently, many of his former students are now practicing throughout the state and the nation. The late PT Joshi, a renowned Panchakarma practitioner, honed [9] his expertise under the tutelage of Tryambak Mahadev Gogate, training alongside the late Shankarsinh Girase at Gogate's hospital in Amaravati (Figure 1). Joshi subsequently established a successful Panchakarma hospital in Dhule and Malegaon, which continues to provide accessible treatment to a large patient population. The hospital also plays a significant role in Ayurvedic education, offering diploma courses in Panchakarma therapy. This institution stands as a testament to Joshi's commitment to both clinical practice and the dissemination of knowledge within the field of Ayurveda [9].

Tryambak Mahadev Gogate passed away on November 26, 1998. Although he is no longer present in this world, his legacy endures through his writings and teachings. His name and contributions remain timeless [4].

## Conclusions

Tryambak Mahadev Gogate's contributions to Ayurveda are groundbreaking, as he seeks to establish a holistic framework that adheres to fundamental principles. His work emphasizes the necessity of appropriate tools for executing diverse Panchakarma techniques and highlights the significance of incorporating modern scientific methodologies to enhance the comprehension and application of Ayurvedic practices.
